# Supplementary material for: Comparative multiomics analysis of cell physiological state after culture in a basket bioreactor
Source: Sci Rep. 2022 Nov 23;12:20161. doi: 10.1038/s41598-022-24687-4 (PMC9686226; doi:10.1038/s41598-022-24687-4)
Supplement: Supplementary file 1 — Supplementary Information 1. [file 41598_2022_24687_MOESM1_ESM.zip › raw data/Transcriptomic raw data/6.Enrichment/1.GO/TE1vsTC1/GSEA/TE1/INTRACELLULAR_ORGANELLE_LUMEN/INTRACELLULAR_ORGANELLE_LUMEN(GO_0070013).html]

Details for gene set INTRACELLULAR\_ORGANELLE\_LUMEN(GO:0070013)[GSEA]


|  || Dataset | readcount.sample |
| Phenotype | sample.cls |
| Upregulated in class | TE1 |
| GeneSet | INTRACELLULAR\_ORGANELLE\_LUMEN(GO:0070013) |
| Enrichment Score (ES) | 0.35683987 |
| Normalized Enrichment Score (NES) | 1.7671927 |
| Nominal p-value | 0.0 |
| FDR q-value | 0.20558044 |
| FWER p-Value | 0.494 |
Table: GSEA Results Summary

  

Fig 1: Enrichment plot: INTRACELLULAR\_ORGANELLE\_LUMEN(GO:0070013)
  
Profile of the Running ES Score & Positions of GeneSet Members on the Rank Ordered List

  

| PROBE | DESCRIPTION   (from dataset) | GENE SYMBOL | GENE\_TITLE | RANK IN GENE LIST | RANK METRIC SCORE | RUNNING ES | CORE ENRICHMENT || 1 | 103236678 | LOC103236678 |  |  | 700 | 6.610 | 0.0287 | Yes |
| 2 | 103242231 | LOC103242231 |  |  | 1531 | 2.404 | 0.0134 | Yes |
| 3 | 103237329 | LOC103237329 |  |  | 1657 | 2.307 | 0.0287 | Yes |
| 4 | 103245731 | ELL3 |  |  | 1736 | 2.263 | 0.0458 | Yes |
| 5 | 103222610 | TAF4B |  |  | 2022 | 2.072 | 0.0518 | Yes |
| 6 | 103241469 | MED12L |  |  | 2190 | 1.990 | 0.0624 | Yes |
| 7 | 103233022 | NUDT21 |  |  | 2321 | 1.926 | 0.0741 | Yes |
| 8 | 103232839 | LOC103232839 |  |  | 2393 | 1.899 | 0.0882 | Yes |
| 9 | 103226096 | HUS1 |  |  | 2431 | 1.885 | 0.1037 | Yes |
| 10 | 103224919 | ELL2 |  |  | 2437 | 1.882 | 0.1205 | Yes |
| 11 | 103223057 | UTP15 |  |  | 2692 | 1.795 | 0.1255 | Yes |
| 12 | 103214372 | GTF2F2 |  |  | 3426 | 1.622 | 0.1074 | Yes |
| 13 | 103222315 | HUS1B |  |  | 3444 | 1.620 | 0.1213 | Yes |
| 14 | 103245539 | GTF2A2 |  |  | 3567 | 1.597 | 0.1304 | Yes |
| 15 | 103242997 | LOC103242997 |  |  | 3716 | 1.571 | 0.1380 | Yes |
| 16 | 103242258 | MED31 |  |  | 3746 | 1.567 | 0.1510 | Yes |
| 17 | 103230666 | TADA1 |  |  | 3875 | 1.544 | 0.1593 | Yes |
| 18 | 103214544 | MRPS31 |  |  | 4089 | 1.516 | 0.1635 | Yes |
| 19 | 103221204 | MRPL47 |  |  | 4098 | 1.515 | 0.1769 | Yes |
| 20 | 103225096 | MEAF6 |  |  | 4171 | 1.506 | 0.1874 | Yes |
| 21 | 103245531 | ICE2 |  |  | 4424 | 1.470 | 0.1894 | Yes |
| 22 | 103244690 | TAF7 |  |  | 4742 | 1.431 | 0.1882 | Yes |
| 23 | 103242815 | MED13 |  |  | 4822 | 1.420 | 0.1976 | Yes |
| 24 | 103239198 | MED13L |  |  | 4848 | 1.417 | 0.2093 | Yes |
| 25 | 103231818 | MED14 |  |  | 4996 | 1.401 | 0.2155 | Yes |
| 26 | 103237327 | RAD21 |  |  | 5038 | 1.397 | 0.2263 | Yes |
| 27 | 103221505 | MED20 |  |  | 5106 | 1.390 | 0.2360 | Yes |
| 28 | 103242814 | INTS2 |  |  | 5353 | 1.363 | 0.2373 | Yes |
| 29 | 103229271 | MED6 |  |  | 5428 | 1.357 | 0.2464 | Yes |
| 30 | 103220076 | MPHOSPH10 |  |  | 5847 | 1.318 | 0.2396 | Yes |
| 31 | 103243867 | MED1 |  |  | 6025 | 1.300 | 0.2435 | Yes |
| 32 | 103223028 | SMARCB1 |  |  | 6407 | 1.270 | 0.2379 | Yes |
| 33 | 103245110 | MAML1 |  |  | 6508 | 1.262 | 0.2449 | Yes |
| 34 | 103220306 | STON1 |  |  | 6604 | 1.252 | 0.2521 | Yes |
| 35 | 103214287 | SUPT20H |  |  | 6634 | 1.249 | 0.2621 | Yes |
| 36 | 103248243 | MED17 |  |  | 7047 | 1.217 | 0.2547 | Yes |
| 37 | 103237144 | LOC103237144 |  |  | 7078 | 1.215 | 0.2644 | Yes |
| 38 | 103225612 | EXOSC10 |  |  | 7203 | 1.205 | 0.2698 | Yes |
| 39 | 103224965 | MED8 |  |  | 7374 | 1.192 | 0.2730 | Yes |
| 40 | 103236289 | MAML3 |  |  | 7443 | 1.186 | 0.2807 | Yes |
| 41 | 103235327 | MED19 |  |  | 7454 | 1.185 | 0.2910 | Yes |
| 42 | 103214895 | POLR3F |  |  | 7489 | 1.182 | 0.3003 | Yes |
| 43 | 103245565 | POLR2M |  |  | 7621 | 1.172 | 0.3050 | Yes |
| 44 | 103225252 | TAF12 |  |  | 7699 | 1.166 | 0.3122 | Yes |
| 45 | 103228222 | COX17 |  |  | 7797 | 1.158 | 0.3184 | Yes |
| 46 | 103225226 | LOC103225226 |  |  | 8014 | 1.142 | 0.3191 | Yes |
| 47 | 103215450 | POLR3D |  |  | 8526 | 1.116 | 0.3063 | Yes |
| 48 | 103214401 | MED4 |  |  | 8713 | 1.103 | 0.3080 | Yes |
| 49 | 103248239 | TAF1D |  |  | 8796 | 1.098 | 0.3143 | Yes |
| 50 | 103226830 | POT1 |  |  | 9506 | 1.053 | 0.2921 | Yes |
| 51 | 103214967 | MED10 |  |  | 9599 | 1.048 | 0.2975 | Yes |
| 52 | 103229444 | GTF2A1 |  |  | 9653 | 1.044 | 0.3046 | Yes |
| 53 | 103223371 | EP300 |  |  | 9745 | 1.038 | 0.3100 | Yes |
| 54 | 103223170 | PES1 |  |  | 9881 | 1.030 | 0.3133 | Yes |
| 55 | 103245617 | LEO1 |  |  | 9893 | 1.029 | 0.3221 | Yes |
| 56 | 103239138 | ERP29 |  |  | 10138 | 1.010 | 0.3204 | Yes |
| 57 | 103231770 | LOC103231770 |  |  | 10273 | 1.006 | 0.3235 | Yes |
| 58 | 103228714 | REC8 |  |  | 10733 | 0.974 | 0.3118 | Yes |
| 59 | 103249020 | TAF4 |  |  | 10825 | 0.968 | 0.3165 | Yes |
| 60 | 103232184 | ORC6 |  |  | 10962 | 0.959 | 0.3191 | Yes |
| 61 | 103229907 | LIN52 |  |  | 11104 | 0.950 | 0.3214 | Yes |
| 62 | 103248950 | MRPL57 |  |  | 11141 | 0.948 | 0.3284 | Yes |
| 63 | 103243722 | FAAP100 |  |  | 11580 | 0.921 | 0.3172 | Yes |
| 64 | 103244860 | MED7 |  |  | 11585 | 0.921 | 0.3254 | Yes |
| 65 | 103222970 | MED15 |  |  | 11789 | 0.907 | 0.3245 | Yes |
| 66 | 103240070 | ORC3 |  |  | 11813 | 0.906 | 0.3317 | Yes |
| 67 | 103234670 | PAF1 |  |  | 11860 | 0.902 | 0.3378 | Yes |
| 68 | 103239694 | MED22 |  |  | 11890 | 0.900 | 0.3447 | Yes |
| 69 | 103234668 | MED29 |  |  | 11948 | 0.897 | 0.3503 | Yes |
| 70 | 103245960 | LOC103245960 |  |  | 11985 | 0.895 | 0.3568 | Yes |
| 71 | 103215919 | RAD21L1 |  |  | 12624 | 0.854 | 0.3360 | No |
| 72 | 103227717 | ALAS1 |  |  | 12871 | 0.841 | 0.3326 | No |
| 73 | 103232147 | MED12 |  |  | 12883 | 0.840 | 0.3398 | No |
| 74 | 103243018 | TEN1 |  |  | 13123 | 0.825 | 0.3366 | No |
| 75 | 103246092 | LOC103246092 |  |  | 14215 | 0.756 | 0.2945 | No |
| 76 | 103225264 | MED18 |  |  | 14582 | 0.734 | 0.2848 | No |
| 77 | 103247164 | MRPS26 |  |  | 15455 | 0.678 | 0.2519 | No |
| 78 | 103227532 | SLX4 |  |  | 15610 | 0.668 | 0.2511 | No |
| 79 | 103233330 | SYCE1L |  |  | 15993 | 0.640 | 0.2398 | No |
| 80 | 103242195 | MED11 |  |  | 16268 | 0.623 | 0.2331 | No |
| 81 | 103234183 | ELL |  |  | 16926 | 0.575 | 0.2089 | No |
| 82 | 103245615 | LOC103245615 |  |  | 17570 | 0.527 | 0.1849 | No |
| 83 | 103220028 | INO80B |  |  | 17649 | 0.519 | 0.1861 | No |
| 84 | 103239112 | RAD9B |  |  | 17658 | 0.519 | 0.1905 | No |
| 85 | 103218667 | RAD9A |  |  | 17676 | 0.517 | 0.1944 | No |
| 86 | 103242473 | MED9 |  |  | 17702 | 0.515 | 0.1980 | No |
| 87 | 103223797 | LOC103223797 |  |  | 17964 | 0.492 | 0.1907 | No |
| 88 | 119619376 | LOC119619376 |  |  | 18347 | 0.455 | 0.1778 | No |
| 89 | 103245959 | LOC103245959 |  |  | 18617 | 0.427 | 0.1696 | No |
| 90 | novel.532 | - |  |  | 22382 | 0.000 | 0.0009 | No |
Table: GSEA details
[plain text format]

  

Fig 2: INTRACELLULAR\_ORGANELLE\_LUMEN(GO:0070013)
  
Blue-Pink O' Gram in the Space of the Analyzed GeneSet

  

Fig 3: INTRACELLULAR\_ORGANELLE\_LUMEN(GO:0070013): Random ES distribution
  
Gene set null distribution of ES for
 **INTRACELLULAR\_ORGANELLE\_LUMEN(GO:0070013)**
